# Exploring CD39 and CD73 Expression as Potential Biomarkers in Prostate Cancer

**DOI:** 10.3390/ph16111619

**Published:** 2023-11-16

**Authors:** Carla Fernanda Furtado Gardani, Eduardo Luiz Pedrazza, Victória Santos Paz, Gabriele Goulart Zanirati, Jaderson Costa da Costa, Roberta Andrejew, Henning Ulrich, Juliete Nathali Scholl, Fabrício Figueiró, Liliana Rockenbach, Fernanda Bueno Morrone

**Affiliations:** 1 Escola de Medicina, Programa de Pós-Graduaҫão em Medicina e Ciências da Saúde, Pontifícia Universidade Católica do Rio Grande do Sul, Porto Alegre 90619-900, RS, Brazil; cfgardani@hotmail.com (C.F.F.G.); lilarockk@gmail.com (L.R.); 2 Laboratório de Farmacologia Aplicada, Escola de Ciências da Saúde e da Vida, Pontifícia Universidade Católica do Rio Grande do Sul, Avenida Ipiranga, 6681, Partenon, Porto Alegre 90619-900, RS, Brazil; eduardo.pedrazza@pucrs.br (E.L.P.); victoria.santos02@edu.pucrs.br (V.S.P.); 3 Instituto do Cérebro da PUCRS, InsCer, Avenida Ipiranga, 6690, Jardim Botânico, Porto Alegre 906010-000, RS, Brazil; gabriele.zanirati@pucrs.br (G.G.Z.); jcc@pucrs.br (J.C.d.C.); 4 Departamento de Bioquímica, Instituto de Química, Universidade de São Paulo, Prof. Lineu Prestes, 748, Butantã, São Paulo 05508-000, SP, Brazil; roberta.andrejew@gmail.com (R.A.); henning@iq.usp.br (H.U.); 5 Programa de Pós-Graduação em Ciências Biológicas: Bioquímica, Instituto de Ciências Básicas da Saúde, UFRGS, Porto Alegre 90035-003, RS, Brazil; juliete.scholl@gmail.com (J.N.S.); fabriciofigueiro@gmail.com (F.F.); 6 Escola de Ciências da Saúde e da Vida, Programa de Pós-Graduaҫão em Biologia Celular e Molecular, Pontifícia Universidade Católica do Rio Grande do Sul, Porto Alegre 90619-900, RS, Brazil

**Keywords:** prostate cancer, ectonucleotidases, CD39, CD73, extracellular vesicles

## Abstract

Prostate cancer (PC) is the most diagnosed tumor in males and ranks as the second leading cause of male mortality in the western world. The CD39 and CD73 enzymes play a crucial role in cancer regulation by degrading nucleotides and forming nucleosides. This study aimed to investigate the expression of the CD39 and CD73 enzymes as potential therapeutic targets for PC. The initial part of this study retrospectively analyzed tissue samples from 23 PC patients. Using the TissueFAXS^TM^ cytometry platform, we found significantly higher levels of CD39—labeling its intensity compared to CD73. Additionally, we observed a correlation between the Gleason score and the intensity of CD39 expression. In the prospective arm, blood samples were collected from 25 patients at the time of diagnosis and after six months of treatment to determine the expression of CD39 and CD73 in the serum extracellular vesicles (EVs) and to analyze nucleotide hydrolysis. Notably, the expression of CD39 in the EVs was significantly increased compared to the CD73 and/or combined CD39/CD73 expression levels at initial collection. Furthermore, our results demonstrated positive correlations between ADP hydrolysis and the transurethral resection and Gleason score. Understanding the role of ectonucleotidases is crucial for identifying new biomarkers in PC.

## 1. Introduction

Prostate cancer is the second leading cause of male mortality from cancer [1]. The treatment of prostate cancer (PC) poses a challenge for biomedical sciences and has a substantial impact on public health given its high incidence and prevalence today. While patients with PC often experience prolonged survival, inadequate risk stratification can lead to the administration of excessive or insufficient treatments, resulting in significant challenges for their well-being [2,3,4,5].

In recent years, extensive research has elucidated various signaling pathways involved in tumor progression, with purinergic signaling emerging as a crucial player [6]. Understanding the tumor microenvironment (TME) has allowed for the identification of the metabolic pathways essential for the survival of tumor cells [7]. Tumoral hypoxia within the TME triggers a multitude of metabolic and immunological changes that facilitate tumor growth and progression [8,9,10]. These changes exhibit proangiogenic effects [11] while simultaneously inducing immunosuppression and impairing effective antitumor responses [12]. Consequently, the TME serves as a site of the production and release of extracellular ATP and adenosine (ADO) [13,14].

The evolving understanding of neoplasms recognizes the pivotal role of the purinergic system. ATP and ADO work together, influencing the TME with both antitumor and protumor effects [15,16]. They function as extracellular ligands for purinergic receptors, impacting cell proliferation, differentiation, and cell death [6]. The purinergic receptor family comprises P1 receptors and P2 receptors, including seven ion channel receptor subtypes (P2Xs) and eight G-protein-coupled receptor subtypes (P2Ys) [17]. Enzymes like CD39 (ectonucleoside triphosphate diphosphohydrolase) convert ATP or ADP into AMP, while CD73 (ecto-5′-nucleotidase) converts AMP to ADO [18].

Previous studies have demonstrated significant differences in nucleotide hydrolysis profiles between patients with prostate cancer, patients with breast cancer, and healthy controls [19,20]. The CD39/CD73-pathway-mediated production of ADO is widely acknowledged as the primary regulatory mechanism employed by regulatory T cells (Tregs). Tregs are a specific subset of CD4+ T cells that play a crucial role in modulating immune responses to antigens [14]. In several cancer types, there is an observed increase in the concentration of Tregs/CD39/CD73 cells, which is directly associated with tumor growth and metastasis [21,22].

In various tumors, including breast cancer, the increased expression of CD73 has been observed. Specifically, in patients with triple-negative breast cancer, higher CD73 expression has been associated with a worse prognosis [23]. Similar associations have been suggested for the outcome of medulloblastomas [24]. In prostate cancer, a study found that elevated CD73 expression is more frequently linked to lymph node metastasis [25]. CD73 emerges as a distinct prognostic determinant in prostate cancer. Its presence within prostate epithelial cells hampers immune surveillance by CD8+ T cells. Even in the neighboring normal prostate epithelium, CD73 expression becomes a potent discriminator between aggressive and indolent prostate cancer forms [26].

Elevated CD39 expression has been observed in various types of solid tumors [27]. Additionally, its expression is notably higher in lymphomas [28,29]. Understanding the mechanisms through which CD39 contributes to tumor-induced immunosuppression is of utmost importance, as targeting this pathway could potentially enhance survival outcomes for certain cancer patients [27].

Various studies suggest that extracellular vesicles (EVs) hold promise as potential markers for the diagnosis and treatment of a wide range of diseases, including cancer [30]. Moreover, CD39 and CD73 have been detected in the EVs released by these cells [31]. Tumor-derived EVs play a crucial role in the transportation of various particles, such as DNA, RNA, mRNA, and microRNA [32]. These vesicles can be found in various body fluids [33,34] and serve for different determinants of tumor progression, allowing the transfer of particles derived from neoplastic cells to healthy cells, among other functions. Previous investigations have showcased the involvement of EVs in the carcinogenesis process of PC as well as their potential as diagnostic or prognostic markers [35].

Understanding the factors influencing tumor progression and establishing a correlation between the expression of ectonucleotidases CD39 and CD73 and the clinical outcomes in cancer are of utmost importance. This study addresses the necessity for accurate risk stratification among PC patients. Furthermore, this study aims to investigate the potential of CD39 and CD73 as predictive biomarkers for the assessment of progression and the treatment response in PC.

## 2. Results

### 2.1. CD39 and CD73 Expression in Tissue Samples from Prostate Cancer Patients

The primary objective of this study was to investigate the association between the tissue expressions of CD39 and CD73 and the Gleason score [36], a crucial clinical parameter used in the diagnosis of prostate cancer, the determination of the clinical stage, and therapeutic decision making for prostate cancer. During the retrospective phase of this study, 23 patients who had been diagnosed with prostate cancer through imaging and biopsies and who subsequently received treatment and follow-up were chosen for analysis. The medical records of these patients were thoroughly reviewed to gather their clinical profiles and to assess their treatment response, disease progression, and disease-free interval (Table 1).

**Figure 1:**
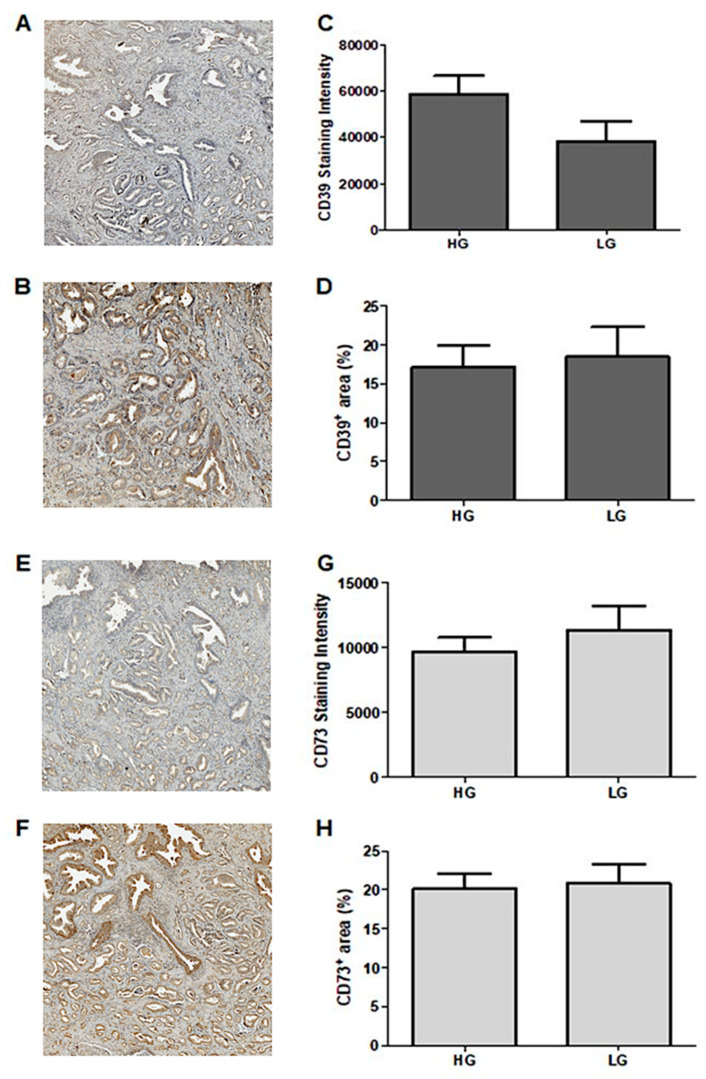
Expression profile of CD39 and CD73 in tissue samples from PC patients. Representative figures of (**A**) negative control and (**B**) positive CD39 labeling. (**C**) CD39 expression profile according to the intensity of positive staining in PC patients. (**D**) CD39 expression profile according to the percentage of positive area in PC patients. Representative images for (**E**) negative control and (**F**) CD73 positive labeling. (**G**) CD73 expression profile according to staining intensity in samples from PC patients. (**H**) CD73 positive area expression profile in samples from PC patients. The results were classified as intermediate and high Gleason scores (**HGs**) and low Gleason scores (**LGs**), as described in the Materials and Methods section. The images were obtained using the TissueFAXS^TM^ microscope and were analyzed using the StrataQuest^TM^ software (TissueGnostics, Vienna, Austria). All images were captured at 400× magnification. The data are expressed as mean ± SEM and were analyzed using Student’s *t*-test with independent samples and the GraphPad Prism 5.1 statistical software, and the results were considered significant for *p*-values.

In this section of the retrospective study, we employed the imaging tissue cytometry technique through Tissue Fax^®^ analyses (TissueGnostics GmbH, Vienna, Austria). The patients were divided into low-Gleason (LG) and intermediate-/high-Gleason (HG) groups, with patients with Gleason scores of six or less belonging to the LG group and patients with a score of seven or more grouped into the HG group. Following the analysis of the samples obtained from the 23 selected patients, we obtained results concerning the proportionate area exhibiting positive CD39 immunostaining. This encompasses both the extent of the positively stained area’s intensity (μm^2^) and the average of the sum intensity per sample (Figure 1A) compared to a negative control (Figure 1B). The average percentage of positive CD39 immunostaining per patient ranged from 1.0% to 35.9%, while the average sum of the positive CD39 immunostaining area ranged from 1228.3 μm^2^ to 55,332.9 μm^2^. Additionally, the sum of the positive CD39 intensity ranged from 5506.1 to 133,079.0 μm^2^.

The analysis demonstrated that the positive staining intensities for CD39 within the HG and LG groups exhibited no significant variation (Figure 1C). Similarly, there was no significant difference in the CD39^+^ percentage area between the two groups (Figure 1D). Subsequently, we performed Spearman’s correlation assessments, determining the correlation between two linear variables. The findings revealed that the correlation between the Gleason score and the percentage area of CD39^+^ yielded a *p*-value of 0.69, while the correlation with the intensity of CD39 positivity in each sample resulted in a *p*-value of 0.06.

The Tissue Fax^®^ analyses conducted for CD73 on the samples from the 23 patients followed the same methodology (Figure 1E,F). The average percentage of positive CD73 immunostaining per patient ranged from 5.9% to 35.5%, while the sum of the positive CD73 immunostaining intensity ranged from 21.2 to 48.0. The patients were also categorized into low-Gleason (LG) and intermediate-/high-Gleason (HG) groups. Similar to the findings related to CD39 expression, our analysis of the positive CD73 immunostaining intensity between the HG and LG groups did not reveal any statistically significant differences (Figure 1G). Furthermore, our results indicated no differences in the percentage of the CD73-positive area between the two groups (Figure 1H). Lastly, we conducted a comparative analysis of the expression profiles of CD39 and CD73 within the two groups, HG and LG. 

### 2.2. Analysis of CD39 and CD73 Expression in Plasma Extracellular Vesicles

In the second phase of our study, we conducted a prospective analysis to establish the baseline and follow-up measurements and subsequently examined their correlation with the degree of response to the administered treatment. In this segment of the study, we enrolled 25 patients diagnosed with prostate cancer and/or biochemical recurrence and a clinical indication for therapeutic management, regardless of the modality, either surgical, radiotherapy, or hormonal therapy. Following the clinical interviews, all the patients underwent the collection of blood samples. Subsequently, six months into the follow-up period, these same patients underwent a second round of blood sample collection to assess their enzymatic activity and expression profiles, which were then correlated with their clinical and laboratory data.

**Figure 2:**
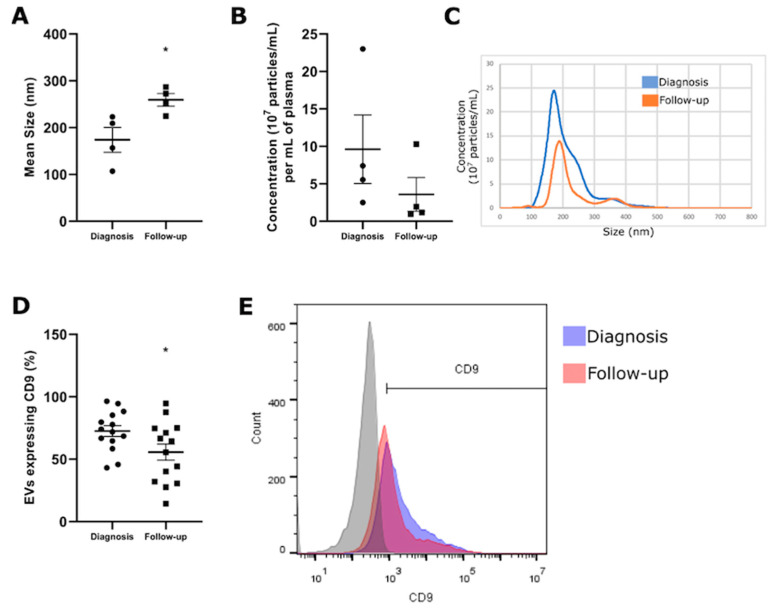
Characterization of size distribution and concentration of the isolated EVs via nanoparticle tracking analysis (NTA). The figure shows the (**A**) mean size, (**B**) particle concentration, (**C**) flow cytometry dot plot, (**D**) CD9 expression, and (**E**) flow cytometry dot plot for EVs isolated from the plasma of patients with CP at diagnosis and follow-up. The grey color represents the negative control. The data are expressed as mean ± SEM and were analyzed using Student’s *t*-test with independent samples in the statistical software GraphPad Prism 5.1. Results were considered significant when * *p* < 0.05.

In order to evaluate the EV release profiles and the expression of the ectonucleotidases in the circulating EVs, we collected peripheral blood, isolated the EVs from the plasma patients, and characterized the size distribution and concentration of the isolated EVs via nanoparticle tracking analysis (NTA). The expression of CD39 and CD73 on the EVs was determined via flow cytometry and costaining with CD9, an EV marker; therefore, the expression of CD39 and CD73 was analyzed only in CD9-positive particles. The EVs were isolated from the plasma of the patients collected at the time of diagnosis and after 6 months of follow-up. The size was consistent with the isolation of small EVs as determined via NTA at both collection times (diagnosis: 197.22 ± 36.45 nm) (follow-up: 259.35 ± 26.95 nm). Interestingly, we obtained smaller EVs at diagnosis compared to follow-up (Figure 2A, *p* < 0.05), with no difference in the particle concentration (Figure 2B,C). This finding suggests a potential alteration in the vesicle composition, possibly involving a shift from smaller EVs, like exosomes, to larger EVs, such as microvesicles and oncosomes. Consistent with the size results, we also observed a decrease in CD9 expression in the follow-up EVs (Figure 2D,E, *p* < 0.05), suggesting that these larger EVs may be enriched with other EV markers. These alterations can exert an influence over the entire microenvironment and the progression of the tumor.

**Figure 3:**
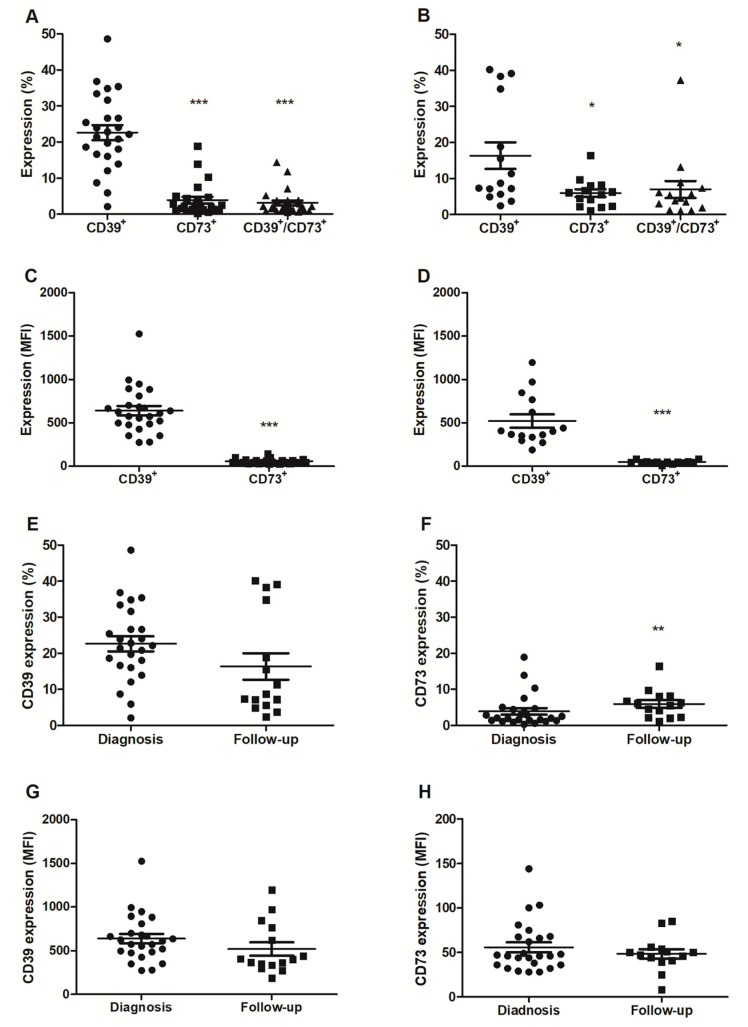
Expression of ectonucleotidases in extracellular vesicles. The percentage of extracellular vesicles with CD39, CD73, and combined CD39/CD73 from the plasma of PC patients at (**A**) diagnosis (*n* = 25) and (**B**) follow-up (*n* = 15). The final values of median fluorescence intensity (MFI) of CD39 and CD73 (**C**) at diagnosis and (**D**) follow-up were described. The comparison of CD39 or CD73 expression at diagnosis and follow-up as percentage (**E**,**F**) or as median fluorescence intensity (MFI) (**G**,**H**). These data were obtained via flow cytometry as described in the Materials and Methods section. Only EVs that showed CD9+ were analyzed. The data are expressed as mean ± SEM and were analyzed using Student’s *t*-test with independent samples in the statistical software GraphPad Prism 5.1. Results were considered significant when * *p* < 0.05, ** *p* < 0.01 and *** *p* < 0.001.

Regarding ectonucleotidase expression, it is worth noting that the results revealed a significant increase in CD39 expression compared to the expression patterns of CD73 (*p* < 0.05) and combined CD39/CD73 (*p* < 0.01) during the diagnosis phase (Figure 3A). However, the same was not observed in the follow-up period (Figure 3B). When considering the median immune fluorescence intensity (MFI), the data indicated a significantly higher expression of CD39 in comparison to CD73 on the plasma-derived EVs both at the diagnosis and after a 6-month follow-up (Figure 3C,D, respectively). Subsequently, we compared the individual expressions of each ectonucleotidase during the two collection times periods (Figure 3E,F). The results indicated an increase in CD73 expression at follow-up compared to during the initial diagnosis period (Figure 3F). Conversely, this pattern was not observed in relation to CD39 expression at both time points, as there was a decrease in expression over 6 months, without statistical significance. Additionally, when assessing CD39 and CD73 based on the measure of fluorescence intensity (MFI) at both collection times, no differences were observed (Figure 3G,H).

Following the initial analyses, the expression levels of CD39 and CD73 (Table 2) were examined at both time points to determine the probability of a positive or negative correlation with each finding associated with the patients’ profiles. Spearman’s correlation was employed, which determines the nonparametric correlation measure ranging from −1 to +1. This method does not require a linear or strictly quantitative relationship between the variables.

Our aim was to establish correlations between CD39 and CD73 expression and the clinical and laboratory data, including the transurethral resection (TUR), clinical stage (CS), Gleason score, and PSA value at diagnosis and follow-up. We obtained interesting findings related to CD73 expression. In the diagnosis phase, we observed a positive correlation between the CD73% and TUR as well as the Gleason score while noting a negative correlation with prostatectomy. Consequently, patients who underwent TUR exhibited a marked increase in CD73% expression (*p* < 0.005) in comparison to those who did not undergo surgical intervention. Moreover, a positive correlation with the Gleason score was observed, implying that a higher score on this scale corresponded to elevated CD73 expression (*p* < 0.05). Conversely, we noted a negative correlation with prostatectomy; thus, patients who underwent this procedure displayed a reduction in CD73% expression (*p* < 0.05). Furthermore, based on the correlation analyses, CD73, during the diagnosis phase (MFI), exhibited a positive correlation with the Gleason score, which reinforces the earlier observed correlation with the CD73%. In relation to the correlations during the follow-up period, the CD73% maintained a positive correlation with the Gleason score (*p* < 0.05) and also with the CS. As a result, higher Gleason scores and/or clinical stages correlated with elevated CD73 expression (Table 2).

### 2.3. Analysis of ATP, ADP, and AMP Hydrolysis Profile in the Plasma

**Figure 4:**
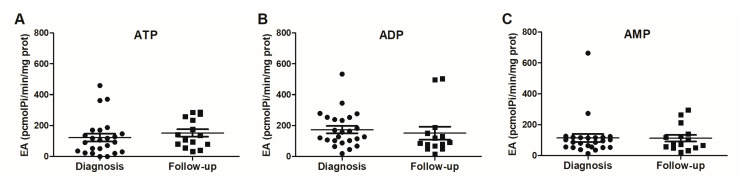
Profile of ATP, ADP, and AMP hydrolysis evaluated in the plasma of patients with PC. The analysis of (**A**) ATPase, (**B**) ADPase, and (**C**) AMPase activities was performed in the plasma of patients with PC at the time of diagnosis (*n* = 24) and follow-up (*n* = 15). The final values are described as ρmol/min/mg of protein. The experiments were performed in triplicate, as described in the Materials and Methods section. The data are expressed as mean ± SEM and were analyzed via Student’s *t*-test with independent samples in the statistical program GraphPad Prism 5.1, with results being considered significant when *p* < 0.05.

The profile of ATP, ADP, and AMP hydrolysis was assessed in the plasma of the patients both at the time of diagnosis and after a 6-month period (follow-up). According to the analysis of the plasma samples obtained pretreatment/at diagnosis, no significant differences were observed in the hydrolysis of ATP, ADP, and AMP among the evaluated patients. Similarly, there were no differences in the analysis of these same nucleotide hydrolysis activities during the follow-up period, which was obtained posttreatment. Subsequently, a comparison of the ATP, ADP, and AMP hydrolysis activities was performed between the two time points: at diagnosis and after 6 months of follow-up (Figure 4). The analysis of the results indicated no significant differences in the ATPase (Figure 4A), ADPase (Figure 4B), and AMPase (Figure 4C) activities when comparing these two collection periods.

Following the initial analysis, we investigated the correlation of the ectonucleotidase activities at both the diagnosis and follow-up time points. As shown in Table 3, it was evident that ADP hydrolysis during the diagnostic phase displayed the most frequent correlations with the clinical data commonly utilized in the medical practice. ADP hydrolysis at diagnosis displayed positive correlations with TUR and the Gleason score while demonstrating a negative correlation among the patients who underwent prostatectomy. Further analysis revealed that the patients subjected to TUR displayed a heightened ADP hydrolysis profile (*p* < 0.01). Conversely, in the patients undergoing prostatectomy, the correlation was negative, implying that those who underwent radical surgical procedures exhibited a reduction in their ADP hydrolysis profile. In terms of the correlation with the Gleason score, it was observed that a higher score corresponded with an increased rate of ADP hydrolysis (*p* < 0.05). Regarding AMP hydrolysis at diagnosis, a significant positive correlation was identified with perineural invasion. This implies that a higher degree of perineural invasion was linked to an elevated profile of AMPase activity (*p* < 0.05).

## 3. Discussion

Prostate cancer stands as one of the most widespread neoplasms and constitutes a major contributor to male mortality worldwide [37,38]. Despite numerous PC patients experiencing prolonged survival periods, the primary cause of death frequently traces back to complications originating from metastasis [38]. In this context, it becomes crucial to identify biomarkers capable of predicting which patients might experience unfavorable clinical outcomes.

The central aim of this study was to explore the potential roles of ectonucleotidases, specifically CD39 and CD73, as biomarkers for the assessment of patient progression and the treatment response. Upon evaluating the expressions of these ectonucleotidases in retrospective tumor samples, a notable expression of CD39 and CD73 was observed. This finding aligns with prior research conducted on various solid tumor types—such as kidney, thyroid, ovary, pancreas, and lung tumors—which consistently showcased a similar pattern of CD39 expression [27]. Additionally, these observations lend support to the notion that CD39 might be intricately involved in the malignant process [39]. Alongside this, we observed nonsignificantly smaller CD39+ areas and a lower intensity of staining. The observed CD73 expression levels in the tissue samples are consistent with the findings of the existing literature, which underline CD73’s tendency to be overexpressed in diverse tumors, like melanoma and breast cancer [23,40]. Another intriguing discovery tied to the CD39 analysis in the tissue samples revealed that the patients with lower Gleason scores exhibited a tendency towards diminished CD39+ percent areas compared to those with higher Gleason scores. Although the intensity of positive CD39 labeling was greater among the patients with lower Gleason scores, the differences were not statistically significant in both sets of results. Furthermore, our analysis of CD73 expression demonstrated no discernible variation in its levels concerning the Gleason score.

Additionally, we noticed a significantly higher expression of CD39 compared to CD73 at the histopathological diagnosis of prostate cancer. This differential expression remained consistently significant regardless of the Gleason score. This particular expression pattern may be attributed to the presence of elevated extracellular ATP (eATP) concentrations, which often stem from conditions like hypoxia, injury, and the action of proinflammatory mediators such as TNF-alpha and IL-6. These elements are hallmarks of the TME [41]. In this process, the presence of heightened eATP levels serves to activate molecular patterns originating from damaged or deceased cells (DAMPs), consequently initiating innate immune responses via the activation of their receptors, primarily Toll-like receptors. This, in turn, promotes processes like phagocytosis and immune cell recruitment, eventually triggering the adaptive immune system as a subsequent step. This orchestrated response aims to combat tumor cells [27]. Consequently, the observed overexpression of CD39 within the TME, which catalyzes the hydrolysis of ATP and/or ADP into AMP, leading to the activation of purinergic receptors, could potentially facilitate tumor progression [6,8,42].

In the prospective arm of our study, we expanded our investigation to explore the expression of CD39 and CD73 within the extracellular vesicles (EVs) and their enzymatic activity in the blood samples both at the time of prostate cancer diagnosis and during the follow-up period. An elevated release of EVs into the bloodstream is linked to numerous pathological processes, including cancer. The presence of specific antigenic markers on the surface of EVs can aid in identifying their cell of origin, facilitating the isolation of vesicles from particular tissue sources, such as tumor tissues [30,34]. Upon analyzing the outcomes concerning the EVs, we observed a prevailing trend of higher CD39 expression compared to CD73 during the diagnosis phase, which corresponded to the pretreatment stage. This heightened CD39 expression persisted not only at diagnosis but also remained evident six months posttreatment in the follow-up phase. This also sustained that high CD39 expression indicates the persistence of a TME that inhibits immune responses through the proinflammatory actions of ATP, creating a favorable environment for the growth of tumor cells. These findings align with previous research demonstrating the inhibitory effects of CD39 on the immune responses mediated by ATP in the TME [43].

It is well established that tumor-cell-derived EVs that overexpress CD39 are also associated with the increased production of ADO, a potent immunosuppressive mediator [31]. Therefore, the combination of elevated CD39 expression and high ADO production in tumor-derived EVs contributes to the establishment of an immunosuppressive microenvironment that promotes tumor growth. Notably, the expression of CD73 in the EVs displayed an increase in the posttreatment follow-up period. It is important to highlight that CD73 plays a key role in the conversion of AMP into ADO [44]. Our results underscore the significance of CD39 overexpression and its association with ADO production in the context of EVs, emphasizing their role in modulating immune responses and shaping the TME to support tumor progression [27].

While this study’s results did not establish a direct correlation between the expression of CD73 in the tissue samples with the clinical and laboratory data, intriguing findings emerged when examining the variables alongside CD73 expression in the EVs. The utilization of a Spearman’s correlation analysis to assess CD39 and CD73 expression within the EVs unveiled several noteworthy correlations. A positive correlation emerged between CD73 expression and the Gleason score, TUR, and the CS. Conversely, a negative correlation was observed with prostatectomy and perineural invasion. Importantly, it is worth noting that all the correlation analyses conducted for CD39 did not yield statistically significant differences. This suggests that CD73 expression in EVs demonstrates a higher frequency of correlations with the prevailing biomarkers employed in clinical practice for PC.

Subsequently, we analyzed the hydrolysis activities of ATP, ADP, and AMP in the plasma of the prostate cancer patients during both the diagnosis phase and the follow-up phase. In light of the heightened expression of CD39 in the tissue samples and EVs coupled with its action characteristics, one might expect the elevated hydrolysis of ATP and ADP at diagnosis in the prospective arm. However, the hydrolysis activities of ATP, ADP, and AMP displayed no significant differences both at diagnosis and follow-up. In the follow-up collection phase, where an alteration in the nucleotide hydrolysis profile or, at the very least, a shift in AMPase activity was expected due to the observed trend of increased CD39 expression in the EVs, our results did not corroborate the earlier reported findings in patients with breast cancer [26]. Specifically, the significant reduction in AMP hydrolysis demonstrated in the plasma of breast cancer patients following oncological treatment, regardless of the specific treatment modality, was not replicated in our study. This discrepancy underscores the complexity and context-specific nature of nucleotide metabolism and its modulation in various cancer scenarios.

Additionally, the Spearman’s correlation analysis at diagnosis unveiled a positive correlation between ADP hydrolysis and TUR and the Gleason score along with a negative correlation with prostatectomy, which further underscores the relevance of CD39’s actions in the context of prostate cancer. Both TUR and prostatectomy involve cellular injury and inflammation, leading to a higher ATP release and thereby stimulating ectonucleotidases to control the circulating nucleotide levels [45]. While there is a scarcity in the literature linking these variables (CD39/ectonucleotidases x TUR and/or prostatectomy), it is known that the CD39/CD73 pathway can be influenced by a range of physiopathological events beyond cancer, including infections, autoimmune diseases, ischemia, infarcts, and more [11]. The correlations between ADP hydrolysis TUR, as well as the Gleason score compared to the prostatectomy outcomes hold significant relevance for future studies.

Furthermore, the correlation analysis of AMP hydrolysis at diagnosis demonstrated a positive correlation with perineural invasion. This suggests that a higher degree of perineural invasion might be associated with an increased profile of AMPase activity. However, the findings related to AMPase activity in this study differ from the previously published results. We observed consistent levels of AMP hydrolysis across the evaluated time points. This contrasts with the study by Gardani and colleagues (2019), which showed elevated AMPase activity in the blood of prostate cancer patients. Their findings suggested that PC patients undergoing radiotherapy exhibit heightened AMP hydrolysis, while patients at lower clinical stages (IIA) present increased ATP hydrolysis compared to those at higher clinical stages (IIB and III). Conversely, AMPase activity was found to be elevated irrespective of the clinical stage of the PC patient [19].

These varied results highlight the intricate and multifaceted nature of nucleotide metabolism and its implications in prostate cancer. However, it is essential to acknowledge that further studies are warranted to validate and substantiate this observation conclusively.

## 4. Patients and Methods

### 4.1. Study Design

The present protocol consisted of two-arm study (Figure S1). The first arm was retrospective, comprising the analysis of CD39 and CD73 expression levels in patient tissue samples taken at the time of diagnosis and the completion of questionnaires based on clinical data from PC patients treated and followed at Hospital São Lucas (HSL) from PUCRS. The second arm of the study was a prospective cohort, and it aimed to determine baseline and follow-up measurements, correlating them with the instituted treatment. The patients with PC were selected at the Oncology and Hematology Center of Cruz Alta (COHCA) at the Santa Lúcia Regional Hospital (HRSL) and the High Complexity Assistance Center in Oncology (CACON) at the São Vicente de Paulo Hospital (HSVP), both located in Cruz Alta/RS, Brazil.

### 4.2. Sample Collection

The retrospective tissue samples were collected from patients with PC who received treatment at the Oncology Service of Hospital São Lucas/PUCRS. Twenty-three paraffin blocks from prostate biopsy samples, which had been previously collected and stored, were used to analyze the expressions of CD39 and CD73 via tissue cytometry.

In the prospective cohort arm, the selected patients were followed for up to 6 months and were treated according to the protocol determined by the attending physician, considering the current indications for treatment plus the current methods of risk stratification and staging. Therefore, patients underwent different modalities of therapeutic interventions, including surgical resection, hormone therapy (HMT), and radiotherapy (RTX), either alone or in combination. Blood samples were collected at diagnosis or biochemical recurrence and after 6 months of follow-up. Analyses were performed to determine the profile of ATP, ADP, and AMP hydrolysis in plasma and the expressions of CD39 and CD73 in plasma extracellular vesicles (EVs).

Inclusion criteria were patients with PC who were over 18 years old with a recent diagnosis of PC or with biochemical recurrence referred for their first visit to the Oncology Services mentioned above. Exclusion criteria were patients with PC, regardless of the EC, who abandoned the proposed oncological treatment and/or did not undergo consultations or complementary exams during the 6-month follow-up period. In the retrospective arm were patients who did not have properly stored paraffin blocks or had insufficient quantities for analysis.

### 4.3. Data Collection

All patients who met the inclusion criteria signed an informed consent form. Questionnaires were also used in both arms of this study to determine which patients met the inclusion criteria for the study as well as to characterize the epidemiological and clinical disease progression profiles.

### 4.4. Immunohistochemistry and Tissue Cytometry

The immunohistochemistry (IHC) assay was performed in tissue samples belonging to the retrospective arm. For this, 5 μm thick sections were subjected to antigen retrieval using Tris/EDTA at pH 9.0 and high temperature; in sequence, they were blocked with 3% ethanol and 5% FBS and were incubated overnight at 4 °C with specific rabbit antihuman CD39 (Abcam, Cambridge, MA, USA, cat#28364) or mouse antihuman CD73 (Santa Cruz, Santa Cruz, CA, USA, cat#32299) followed by respective antirabbit or antimouse horseradish peroxidase secondary antibodies (Alomone Labs^TM^, Jerusalem, Israel). Afterwards, samples were incubated with 3,3′-diaminobenzidine (DAB) (Novolink Chromogen, Leica Biosystems, Inc., Buffalo Groove, IL, USA) for 5 min followed by hematoxylin counterstaining. Imaging cytometry scanning of IHC-stained tissue slices was performed using TissueFAXS™ Cytometry platform (TissueGnostics GmbH, Vienna, Austria), and quantification was performed using StrataQuest^TM^ software (TissueGnostics). Cell numbers and areas were determined by using cellular masks and via segmentation analysis [46,47]. CD39 or CD73 immunostaining was marked in brown, while nuclei were distinguished in blue; the software detects different color shades and builds a mask for nuclei (blueish) and other mask(s) for the cytoplasm and membrane of cells, which contain CD39 or CD73 staining, by detecting brownish shades. The average percentage (%) of positive staining of the samples for each of the ectonucleotidases was determined via cytometry analysis; the average of the sum of intensity per sample area was also determined. The data are presented as area of the sample with positive staining.

### 4.5. Extracellular Vesicle Analysis

Patients selected for the prospective arm of this study underwent blood collection (4 mL) before and after treatment, with a 6-month interval between them. Blood samples were collected in BD heparin tubes (BD Bioscience, Franklin Lakes, NJ, USA) and were kept refrigerated until centrifugation, which was performed for 10 min at 5000 rpm or 12 min at 4000 rpm, followed by storage at −80 °C. Plasma samples were prepared for EV isolation following the method described by Jiang et al. (2014) [48]. Briefly, plasma samples were centrifuged at 10,000× *g* for 30 min at 4 °C and were then subjected to ultrafiltration at 0.22 μm followed by ultracentrifugation at 100,000× *g* for 90 min at 4 °C. The EVs were resuspended in PBS, and their proteins were quantified using the BCA kit [49] from ThermoFisher. For nanoparticle tracking analysis (NTA), a NanoSight instrument (LM10, NanoSight Ltd., Malvern Panalytical, Malvern, Worcestershire, UK) and NTA 2.0 (Analytical Software Malvern Panalytical, Malvern, Worcestershire, UK) were used to analyze the individual particles in the formulations after dilution (1:5000) by examining Brownian motion in real time via a CCD camera.

Flow cytometry was used to determine the expressions of CD9, CD39, and CD73 on the EVs, as described by Suárez et al. (2017) [50]. Briefly, latex beads (4 μm) able to bind to EVs were used (ThermoFisher, cat#A37304). Initially, bead-coupled EVs were stained with primary Ab CD9 (1:200, clone was M-L13, BD Biosciences, San José, CA, USA, cat#555370) for 30 min (RT), were washed with blocking buffer (PBS + 2% SFB), and were stained with goat antimouse Alexa Fluor™ 488 (1:100, ThermoFisher, cat#A-11001) for 30 min at 4 °C. After the incubation time, samples were washed twice with blocking buffer followed by staining with anti-CD39-APC (1:30, clone was TU66, BD Biosciences, cat# 560239) and anti-CD73-PE (1:30, clone was AD2, BD Biosciences, cat#550257) for 30 min at 4 °C. Afterwards, bead-coupled EVs were washed twice and analyzed using BD Accuri^TM^ flow cytometer and C6 software (BD Biosciences, San José, CA, USA). CD39/CD73 expression was analyzed only in CD9-positive EVs.

### 4.6. Analysis of ATP, ADP, and AMP Hydrolysis

Patients selected for the prospective arm of this study underwent blood collection (4 mL) before and after treatment, with a 6-month interval between them. Blood samples were collected in plastic tubes with heparin (green tube) and were kept refrigerated until centrifugation, which was performed for 10 min at 5000 rpm or 12 min at 4000 rpm, followed by storage at −80 °C until enzymatic analysis. The samples were kept at a controlled temperature during transportation between the referenced services and the Applied Pharmacology Laboratory at PUCRS. To determine the nucleotide hydrolysis activity in plasma, samples (50 uL of plasma) were added to Eppendorfs containing 90 uL of 250 mM Tris-HCl, pH 8.0, kept on ice. Then, the samples were preincubated for 10 min in a water bath at 37 °C. Then, incubation began with the addition of 60 uL of substrate (10 mM ATP, ADP, or AMP; final concentration 3 mM). After 50 min, the reaction was stopped by adding 200 uL of 10% TCA and transferring the tube to ice. After incubation, the samples were centrifuged at 15,000 rpm for 10 min, and 20 uL of the supernatant was added to 1 mL of fresh malachite green reagent. Then, 20 min later, the absorbance of the samples was read in a spectrophotometer at 630 nm. All samples were run in triplicate, and hydrolysis controls were created; they received 50 uL of plasma only after the reaction was stopped. The malachite green method determined the amount of Pi released, and the amount of protein was determined by Coassie blue, resulting in the specific activity of the enzyme in picomoles of Pi/min/mg of protein, according to the incubation protocol described by Moritz et al. (2017) and Chan et al. (1986) [51,52].

### 4.7. Statistical Analysis

The correlations of patients’ clinical data were done using Spearman’s correlation in SPSS 25.0 program. Spearman’s correlation determines the measure of nonparametric dynamics ranging from −1 (negative correlation) to +1 (positive correlation). Differences were considered significant when *p* < 0.05.

All other assay results are expressed as mean ± standard deviation. Statistical analyses were performed using *t* test or one-way ANOVA followed by post hoc Tukey in GraphPad Prism 5.1 (San Diego, CA, USA), which was also used for graphics production.

## 5. Conclusions

This study unveiled the expression of CD39 and CD73 within retrospective prostate tumor samples. Additionally, it demonstrated a higher expression of CD39 compared to CD73 in the plasma extracellular vesicles either individually or in combination with CD73. Intriguingly, ADP hydrolysis at diagnosis displayed positive correlations with transurethral resection and the Gleason score, while a negative correlation was observed in the patients who underwent prostatectomy. The present study has shown intriguing results with the analysis of the tissue samples as well as the examination of ectonucleotidase expression in the extracellular vesicles and the hydrolysis activities of the nucleotides. However, it is imperative to conduct studies on new cohorts of prostate cancer patients, preferably with larger sample sizes and longer follow-up periods. In summary, the identification of predictive markers in prostate cancer holds immense significance, emphasizing the critical role of understanding the involvement of ectonucleotidases in the development of this disease.

## Figures and Tables

**Table 1:** Clinical and pathological data of prostate cancer patients in the retrospective arm.

Characteristics	N (23)	%
Age (Years)		
Mean	65.4	
Range	48–78	
PSA		
<10	15	65.5
10–20	6	26.1
>20	2	8.7
Gleason Grade *		
Low grade	7	30.5
Intermediate grade	12	52.2
High grade	4	17.3
Histology		
Adenocarcinoma	23	100
Clinical Stage (CS) **		
CS I/EC IIA	7	30.5
CS IIB/EC IIC	11	47.9
>CS IIIA	5	21.6
Transurethral Resection—TUR		
Yes	3	13.1
No	20	86.9
Radical Prostatectomy		
Yes	12	52.2
No	11	47.8
Radiotherapy		
Yes	18	78.3
No	5	21.7
Hormonotherapy		
Yes	9	39.1
No	14	60.9

* Gleason score—low/well-differentiated tumors (Gleason of ≤ 6), intermediate/moderately differentiated tumors (Gleason of 7), and high/poorly differentiated tumors (Gleason of 8–10). ** Clinical stage (CS)—early-stage disease (CS I and IIA), intermediate-stage disease (CS IIB and C), and locally advanced disease (>III).

**Table 2:** Nonparametric data analysis of CD39 and CD73 expression in EVs and clinical and laboratory data at diagnosis and follow-up.

	CD39 Diagnosis		CD39 Follow-Up		CD73 Diagnosis		CD73 Follow-Up	
**N**	(%)	(MFI)	(%)	(MFI)	(%)	(MFI)	(%)	(MFI)
Age								
Mean (68.6)	−0.045	0.046	−0.322	−0.344	−0.064	−0.144	−0.199	−0.255
Range (52–94)								
TUR								
Yes (17)	−0.107	−0.048	0.327	0.196	0.631 **	0.197	0.491	0.344
No (8)								
Prostatectomy								
Yes (8)	−0.83	−0.131	−0.098	−0.131	−0.553 **	−0.197	−0.229	−0.115
No (17)								
Gleason Score								
<6 (5)								
7 (13)	−0.017	0.048	0.342	0.197	0.455 *	0.170	0.594 *	0.318
8–10 (7)								
CS								
I/IIA (9)	0.133	0.188	0.015	−0.165	0.392	0.173	0.559 *	0.065
IIB/IIC (14)								
III (3)								
Perineural Invasion								
Yes (18)	−0.334	−0.383	−0.182	0.045	−0.192	−0.445 *	0.091	−0.023
No (7)								
PSA								
<10 (18)	0.166	0.174	0.354	0.343	0.083	0.171	−0.221	0.204
10–20 (4)								
>20 (3)								

Gleason scale—low/well-differentiated tumors (Gleason of 2–6), intermediate/moderately differentiated tumors (Gleason of 7), and high/poorly differentiated tumors (Gleason of 8–10). Clinical stage (CS)—early-stage disease (CS I and IIA), intermediate-stage disease (CS II B and C), and locally advanced disease (>III). Percentage (%) and median fluorescence intensity (MFI). Spearman’s correlation was used, which determines the nonparametric correlation measure ranging from −1 (negative correlation) to +1 (positive correlation). * *p* < 0.05, and ** *p* < 0.005.

**Table 3:** Correlation of the ectonucleotidase activities with clinical and laboratory data at diagnosis and follow-up.

	Diagnosis			Follow-Up		
	ATP Hydrolysis	ADP Hydrolysis	AMP Hydrolysis	ATP Hydrolysis	ADP Hydrolysis	AMP Hydrolysis
Age	0.202	0.300	−0.037	−0.245	−0.141	0.048
TUR	0.217	0.549 **	0.013	−0.393	0.033	−0.229
Prostatectomy	−0.013	−0.421 *	0.051	0.360	−0.065	−0.065
Gleason Score	0.254	0.427 *	0.397	−0.097	0.220	0.147
CS	0.246	0.179	0.241	0.094	0.320	0.335
Perineural Invasion	−0.049	0.125	0.431 *	0.454	0.409	0.136
PSA	0.010	−0.023	−0.171	−0.182	−0.357	0.207

Spearman’s correlation, which determines a nonparametric correlation measure ranging between −1 (negative correlation) and +1 (positive correlation). * *p* < 0.05. ** *p* < 0.005.

## Data Availability

The data presented in this study are available on request from the corresponding author. The data are not publicly available due to the Brazilian Lei Geral de Proteção de Dados (LGPD).

## Supplementary Materials

The following supporting information can be downloaded at https://www.mdpi.com/article/10.3390/ph16111619/s1. Figure S1: Schematic representation of the study design. Figure S2: Expression of CD39 and CD73 percentages and median fluorescence intensity in extracellular vesicles extracted from prostate cancer patients.
