# Supplementary material for: Exploring CD39 and CD73 Expression as Potential Biomarkers in Prostate Cancer
Source: Pharmaceuticals (Basel). 2023 Nov 16;16(11):1619. doi: 10.3390/ph16111619 (PMC10675019; doi:10.3390/ph16111619)
Supplement: Supplementary file 1 [file pharmaceuticals-16-01619-s001.zip › pharmaceuticals-2617083-supplementary.pdf]

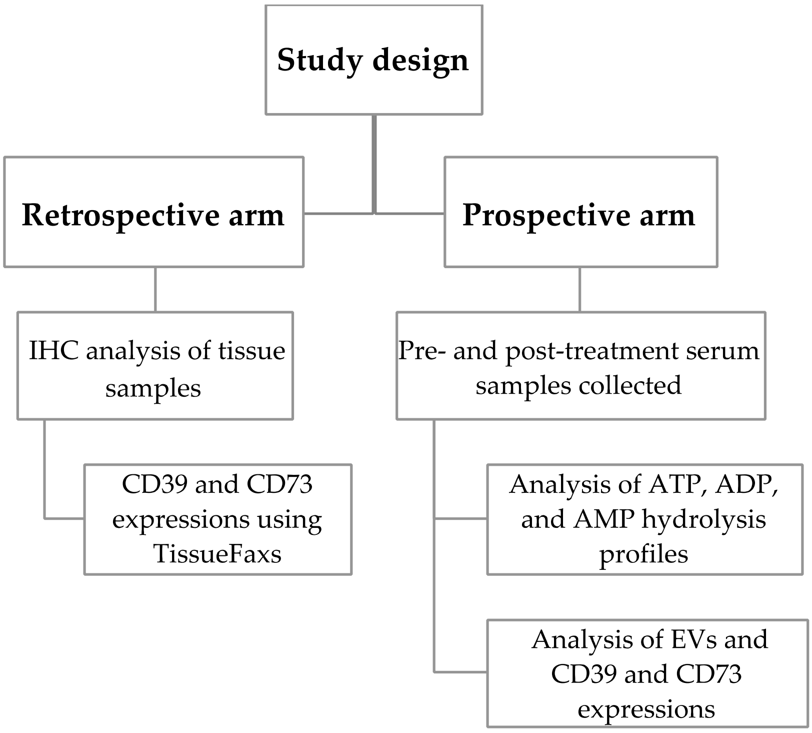

Figure S1. Schematic representation of the study design.

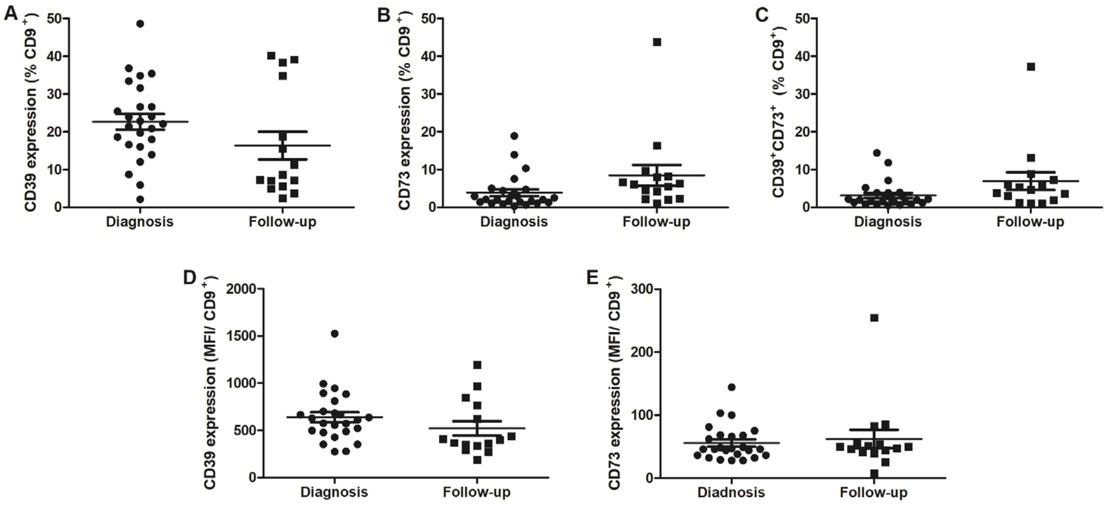

Figure S2. Expression of CD39 and CD73 percentages and median fluorescence intensity in extracellular vesicles extracted from prostate cancer patients. Plasma from blood collection at diagnosis and after 6 months of follow-up. **(A)** Expression of C39 percentage, **(B)** C73 percentage, **(C)** combined expression of CD39 and CD73, **(D)** expression of CD39 median fluorescence intensity **(E)** CD73 median fluorescence intensity in EVs extracted from patient plasma from blood collection at diagnosis and after 6 months of follow-up/post-treatment. Only CD9+ EVs were analyzed, and the final values were described as a percentage (%) and median fluorescence intensity (MFI), as described in the Materials and Methods section. Data were expressed as mean ± SEM and analyzed by one-way independent samples t-test in GraphPad Prism 5.1 statistical software.
